# How minimal executive feedback influences creative idea generation

**DOI:** 10.1371/journal.pone.0180458

**Published:** 2017-06-29

**Authors:** Hicham Ezzat, Anaëlle Camarda, Mathieu Cassotti, Marine Agogué, Olivier Houdé, Benoît Weil, Pascal Le Masson

**Affiliations:** 1 Center for Management Science, Chair TMCI, Mines ParisTech, Paris, France; 2 CNRS Unit 8240, Laboratory for the Psychology of Child Development and Education, Paris Descartes University and Caen University, Paris, France; 3 Institut Universitaire de France, Paris, France; 4 HEC Montréal, Montréal, Canada; University of Georgia, UNITED STATES

## Abstract

The fixation effect is known as one of the most dominant of the cognitive biases against creativity and limits individuals’ creative capacities in contexts of idea generation. Numerous techniques and tools have been established to help overcome these cognitive biases in various disciplines ranging from neuroscience to design sciences. Several works in the developmental cognitive sciences have discussed the importance of inhibitory control and have argued that individuals must first inhibit the spontaneous ideas that come to their mind so that they can generate creative solutions to problems. In line with the above discussions, in the present study, we performed an experiment on one hundred undergraduates from the Faculty of Psychology at Paris Descartes University, in which we investigated a minimal executive feedback-based learning process that helps individuals inhibit intuitive paths to solutions and then gradually drive their ideation paths toward creativity. Our results provide new insights into novel forms of creative leadership for idea generation.

## Introduction

Fixation effects [1] have always been recognized as among one of the most important barriers to creativity. Over the past decades, numerous cognitive science studies have underlined the obstructive function against creative ideation of the spontaneous activation of known solutions and knowledge in individuals’ minds. These studies have demonstrated that previously acquired knowledge in individuals’ minds fixate them and consequently restrain their aptitude for the generation of creative ideas [2].

Numerous psychologists have been interested in demonstrating fixation effects [1, 3, 4]. One classical task illustrating such effects is the “two cord problem” [3]. Participants are given two cords that are tied to the ceiling and a pair of pliers. The participants are then asked to tie the free ends of these two cords together with the knowledge that the cords are short and cannot be held in the hands at the same time in a manner in which one could easily tie them together. One solution to this problem is to simply tie the pliers to one of the cords to form a pendulum that will swing to enable the reaching of the second cord. In this experiment, most participants are fixated on their proper knowledge of pliers and their conventional uses and do not consider the alternative use of the pliers to form a pendulum.

Over the past years, the field of design science has been very useful to the modeling and precise identification of these cognitive biases to creativity. Indeed, Concept-Knowledge (C-K) theory [5] is well renowned as a tool to not only force designers’ reasoning to succeed in overcoming fixation effects [6] but is also recognized to aid the generation of ideas that are inside or outside of existing paradigms [7]. This theory distinguishes between a fixation path that is based on the spontaneous activation of knowledge (inside fixation) and an expansive path that is based on the activation of less accessible knowledge (outside fixation) and consequently offers a method to characterize different paths of solutions in addition to the knowledge bases associated with these solutions.

Using this C-K-based cartography of solutions, interdisciplinary studies that mix human cognition with design theory have been able to develop smart lock-in methodologies to overcome fixation effects. These studies have demonstrated the stimulating role of expansive examples, i.e., ideas and solutions that are outside fixation effects, in elevating the creative generation capacities of individuals [8]. The authors utilized a classical creative ideation task that consists of proposing the maximum number of solutions to ensure that a hen’s egg dropped from a height of ten meters does not break. Using an existing database of solutions created over the last five years [8], the authors revealed that 81% of the solutions belonged to three categories of “restrictive” solutions within the fixation path (i.e., damping the shock, slowing the fall, and protecting the egg). However, only 19% of the solutions were “expansive” solutions, i.e., solutions that were outside of the fixation path (for instance, solutions implemented before and after the fall, the use of a living device, and the use of the intrinsic properties of the environment). The authors then demonstrated that, when the participants were given a creative example (outside the fixation path) prior the task, they proposed more original solutions. Similarly, these studies also emphasized the obstructive role of restrictive examples, i.e., ideas and solutions that were inside the fixation path, to the creative generation process. These studies were performed with participants with different backgrounds (i.e., students, psychologists, engineers, and designers) [9] and different ages [10, 11] and have noticeably confirmed the negative role of restrictive examples (i.e., examples within the fixation path) on the fluency and originality of the proposed solutions to the same creative task.

Developmental psychology theorists have analyzed the problem at the reasoning level and realized that thinking outside the box may also require first resisting what is inside the box. Indeed, these scholars have investigated the problem of cognitive biases at the reasoning processes level and have underscored the critical role that could be played by inhibitory control of the fast and intuitive system of reasoning in overcoming heuristics in certain cases [12–14]. Based on the dual-process theory of reasoning comprising both an intuitive system (system 1) and an analytic system (system 2) [15, 16], these authors have proposed a third system termed “cognitive inhibition” (system 3) [13]. The latter system plays the role of inhibiting the fast and intuitive system (system 1) to release the slow and analytic system (system 2). Along these specific lines, recent works have linked these above-mentioned findings with the context of cognitive biases to creativity. Considering that the difficulty in generating creative ideas might result from individuals’ failures to inhibit spontaneous responses that come to mind and lead them to fixate on certain knowledge, these authors have proposed an analogical model of reasoning in creativity situations that they termed the “dual-process model of creativity” [17]. Similarly, these works argue that the abilities of individuals to resist the spontaneous activation of design heuristics by inhibiting inappropriate ideas is a crucial factor in the generation of creative ideas [18–20].

In line with the above ideas, in the current paper, we propose a learning process that can be implemented to guide individuals’ systems of reasoning for creativity. More precisely, with the help of design theories, such as the C-K theory [21], in the present study, we analyzed the roles of feedback processes in i) the inhibition of obvious solutions to a particular creativity task and ii) the gradual forcing of individuals’ reasoning to explore and activate novel and creative ideas and solutions to problems.

The concept of feedback is widely used in different domains, and its definition varies significantly depending on discipline [22]. Feedback can be described as the control of a process based on its results, i.e., the output of an action is returned to modify the subsequent action. Feedback is an efficient instrument in the control and regulation of individuals’ performance in real-time and is extensively used in learning processes.

Few studies have been devoted to the relationship between feedback and creativity. Most researchers have examined feedback from a very broad perspective. These researchers have investigated the influence of evaluative information on creative performance and argued that it could have a strong influence on enhancing creative processes [23]. Indeed, these studies have underscored the importance of being exposed to others’ ideas and perspectives in the stimulation of the generation of creative ideas. Other studies have noted that feedback can significantly help to regulate individuals’ creative performances [24]. Moreover, other findings have argued that delivering negative and controlling feedback to individuals can damage their creative performance, and in contrast, the delivery of constructive or developmental feedback can exert a positive influence on creativity [24–28].

In the domain of reasoning, Moutier and Houdé [29–31] developed a training paradigm that involves explicit executive feedback regarding various reasoning biases. Using a classical pre-test/training/post-test design, the efficiency of this training procedure is indexed by comparing the post-test performance with the performance in the control training with the logic that the latter only differs due to the absence of executive feedback. Therefore, the specificity of the executive training lies in the presence of executive feedback, such as “we’re falling into a trap! (. . .)” or “The goal here is not to fall into the trap (. . .)”. The words “not to fall into the trap” in this training procedure are introduced to provoke a tendency to reject the biased strategy. Although the reasoning biases were found to be very high, the results revealed that only the executive training improved the subjects’ metacognitive ability to overcome classical reasoning biases, such as the conjunction fallacy and the matching bias, during deductive reasoning [29]. In other words, this study emphasized the near transfer effect by confirming that the executive training could be transferred to structurally similar tasks. This experimental design was also applied during a brain imaging study, and the results revealed a reconfiguration of neural activity that correlated with the near executive transfer effect in the domain of deductive reasoning [32]. The results revealed clear shift in neural activity from the posterior part of the brain prior to executive training (i.e., when the participants’ responses were biased by the use of system 1) to the prefrontal portion after training (i.e., when they became able to inhibit the system 1 intuitive response and provide the correct answer via the use of system 2). Altogether, these findings demonstrated that executive feedback can provoke the inhibition of strongly intuitive wrong answers [33] and provided the first insights into the neuropedagogy of reasoning [34].

Despite the contributions made to the literature of creativity and the importance of studying the influence of feedback on ideation from this above-mentioned relatively broad perspective, to the best of our knowledge, no previous studies have focused on the influence of executive feedbacks from a deeper perspective from which minimal feedback might control individuals’ ideations during real-time processes to guide them outside of fixation.

In the present study, we propose a minimal executive feedback-based learning model that could guide individuals’ idea generation paths whether inside fixation, i.e., a conceptual space associated with the fixation effect, or in expansion, i.e., a conceptual space associated with concepts outside of fixation. In other words, we were interested in modeling a learning process that can guide individuals’ ideation paths toward certain types of ideas and solutions whether they are restrictive, i.e., do not change an object’s definition or attributes, or expansive, i.e., transform an object’s definition and identity [8].

Therefore, the aim of the present study was to examine how minimal executive feedback influences individual ideation in real-time. To achieve this aim, participants were asked to solve a creative task (i.e., the egg task) and were provided with minimal executive feedback after each generated solution.

Critically, the executive feedback was either congruent or incongruent with the creative aim of the egg task. In the congruent executive feedback condition, the feedback suggested that the participants “search for another path” when the proposed solution belonged to the fixation path and “continue in this path” when the solution belonged to the expansive path. In the incongruent feedback condition, the feedback suggested that the participants “continue in this path” when the proposed solution belonged to the fixation path and “search for another path” when the solution belong to the expansive path.

We reasoned that if creative idea generation requires the inhibition of the intuitive path to the solution that leads to the fixation effect, as posited by the dual process model of creativity and the C-K theory of design, then the executive feedback should have affect the participants’ performances in the egg task relative to a control condition that involved no instructive feedback (i.e., “I confirm the receipt of your idea”). Specifically, the congruent executive feedback should improve performance by facilitating the inhibition of ideas within fixation and stimulating the exploration of ideas in expansion, whereas the incongruent executive feedback should impair performance by interfering with the inhibition of uncreative ideas that lead to fixation and stimulating the exploration of ideas within the fixation path.

## Experiment 1

### Method

#### Participants

Sixty undergraduates from Paris Descartes University participated in this study (32 men, mean age = 20.5 years, SD = 2.62). Each participant was randomly assigned to one of the three following experimental conditions: congruent executive feedback (n = 20; 13 men), incongruent executive feedback (n = 20; 12 men), and a control group that received neutral feedback (n = 20; 7 men). ANOVA and chi-squared analyses indicated that the mean ages (F(1,57) < 1) and gender distributions (χ2 = 1.70, p = 0.12) did not differ significantly between the groups. All the participants provided written consent and were tested in accordance with national and international norms governing the use of human research participants. The institution that granted permission for the following experiments is the faculty of psychology of the University of Paris Descartes.

#### Procedure

The participants sat alone in an experimental room in front of a computer and were asked to wait for the experimenter to contact them via a text (written) chat conversation using Skype. The experimenter initiated the chat conversation and provided the following initial brief to the subject: “design a process that allows by which a hen’s egg that is dropped from a height of ten meters does not break”. Each subject was then instructed by the experimenter to write down, in the chat conversation, the maximum number of original ideas they could generate to solve this problem. The task duration was set to 10 minutes per participant.

Using an existing database of solutions that was collected over the last five years [8], two experimenters were trained before the experiment to identify whether a generated idea belonged to the fixation paths (which included damping the shock, slowing the fall, and protecting the egg) or were outside of those paths (for instance, interventions implemented before or after the fall, the use of a living device, the use of the intrinsic properties of the environment, etc.). Table 1 lists the categories of solutions to the hen’s egg task according to the database.

**Table 1:** Categories of solutions to the egg task [8].

Categories	Example of Solutions
Damping the shock	Place a mattress at the reception
Protecting the egg	Pack the egg with bubble wrap
Slowing the fall	Hang the egg to a parachute
Interrupting the fall	Catch the egg with a net
Acting before the fall	Drop the egg at a height of 11 m
Acting after the fall	Replace the broken egg with an unbroken one
Using a living device	Train an eagle to take down the egg
Modifying the properties of the egg	Freezing the egg
Using the natural properties of the egg	Drop the egg on its most robust axis
Using the properties of the environment	Drop the egg at zero gravity

The participants in the control group received neutral feedback that simply acknowledged the reception of an idea generated by the subordinate and awaited the next idea. For the participants in the congruent executive feedback group, if the generated idea was in the fixation path, the feedback provided was “search for another path”; in contrast, if the generated idea was in the expansion path, the provided feedback was “continue in this path”. In contrast to the congruent executive feedback group, for the participants in the incongruent executive feedback group, if the generated idea was in the expansion path, the provided feedback was “search for another path”; in contrast, if the generated idea was in the fixation path, the provided feedback was “continue in this path”.

#### Results

To examine whether the numbers of proposed solutions (i.e., fluency) within the fixation path (fixation) and outside the fixation path (expansivity) varied according to the experimental conditions, we conducted a repeated-measures analysis of variance (ANOVA) with the experimental condition (congruent; control and incongruent) as a between-subjects factor and the category of solution (fixation vs. expansion) as a within-subjects factor, and we used the partial eta squared (η_p_^2^) and Cohen’s d to examine the effect size.

**Fig 1:**
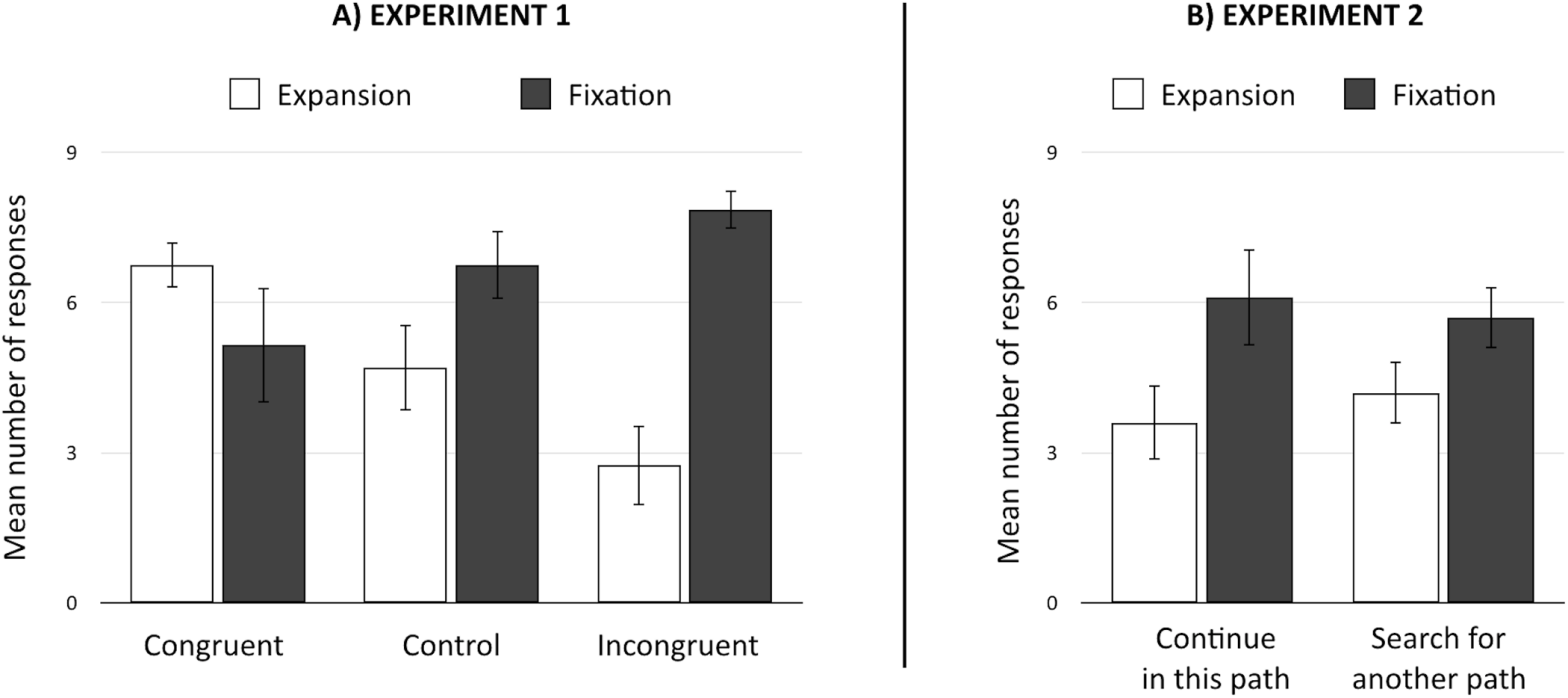
Mean number of solutions according to the experimental condition (A: Congruent/Control/Incongruent; B: Continue in this path/Search for another path) and the type of solution (Expansion/Fixation).

This analysis revealed a main effect of the solution category (F(2, 57) = 9.49, p < .005, η_*p*_^*2*^ = .14, Power = .86) that indicated that the participants provided more solutions in the fixation path than in the expansion path. There was no main effect of the experimental condition (F(2, 57) < 1). However, there was a significant experimental condition x category of solution interaction (F(2,57) = 10.4, p < 0.001, η_*p*_^*2*^ = .27, Power = .99, see Fig 1A).

One-tailed planned comparisons were corrected with a Holm–Bonferroni procedure for analyses of the number of solutions within the fixation path and within the expansion path separately. Results revealed no significant difference between the number of solution within the fixation path in the control group (M = 6.75, SD = 3.85) and those in the congruent group (M = 5.15, SD = 2.06; F(1/57) = 2.42, *p*_*corr*_ = .12, d = .52). In addition, there was no significant difference between the number of solution within the fixation path in the incongruent group (M = 7.85, SD = 3.56) compared to the control group (M = 6.75, SD = 3.85; F(1/57) = 1.14, *p*_*corr*_ *=* .29, d = .30). Interestingly, participants proposed fewer solutions within the fixation path in the congruent group (M = 5.15, SD = 2.06) than participants in the incongruent group (M = 7.85, SD = 3.56; F(1/57) = 6.89, *p*_*corr*_ = .03, d = .92)

Critically, the participants in the control group (M = 4.7, SD = 3.04) proposed fewer solutions in the expansive path than did those in the congruent group (M = 6.75, SD = 5.12; F(1/57) = 3.88, *p*_*corr*_ = .05, d = .49). Additionally, the participants in the control group (M = 4.7, SD = 3.04) proposed more solutions in the expansive path than did those in the incongruent group (M = 2.75, SD = 1.71; F(1/57) = 3.51, *p*_*corr*_ = .032, d = .79). Finally, the participants in the congruent group (M = 4.7, SD = 3.04) proposed more solutions in the expansive path (M = 6.75, SD = 5.12) than did those in the incongruent group (M = 2.75, SD = 1.71; F(1/57) = 14.79, *p*_*corr*_ = .0005, d = .1.05).

#### Discussion

The aim of the present study was to examine the influence of a minimal executive feedback-based learning process on the performance of an individual ideation task in real-time to explore how such feedback could guide individuals’ creative reasoning. Three major findings emerged from this investigation as follows: 1) congruent executive feedback increases individuals’ idea generation within the expansive path; 2) incongruent executive feedback has the opposite effect; and 3) critically, incongruent executive feedback had a weaker effect on creative performance than did congruent executive feedback.

Our results demonstrated that our minimal executive feedback-based learning process could be implemented to gradually force individuals’ reasoning to explore and activate novel and creative ideas and solutions to problems. This stimulatory effect of the congruent executive feedback extends previous findings regarding the influence of training paradigms involving explicit executive feedback on various reasoning biases [29–31]. Indeed, these studies have consistently reported that executive training can greatly improve individuals’ metacognitive abilities to overcome classical reasoning biases, such as the conjunction fallacy and the matching bias, during deductive reasoning. Moreover, our results are also coherent with those of previous studies that have been performed on the neuropedagogy of reasoning [34] and demonstrated that minimal executive feedback can clearly provoke the inhibition of strongly intuitive wrong answers [33].

While our findings support the dual systems model of creativity, one limitation of the present study might be that depending on the experimental condition, participants might simply interpret the feedback “search for another path” and “continue in this path” as meaning something along the lines of “be more creative” and “be less creative” respectively. Given that the same feedback were used in both the congruent and the incongruent conditions this alternative explanation seems less likely. Nevertheless, to determine whether the stimulation effect of the congruent feedback condition arise from the interpretation of the instruction “search for another path” as “be more creative” and the instruction “continue in this path” as “be less creative”, the influence of these specific feedback regardless of the response provided by the participant were examined in a second experiment. We reasoned that if participants interpret the instructions as mentioned below, they should generate more creative responses when they receive “search for another path” feedback after each generated solution, and fewer creative responses when they receive “continue in this path” feedback.

## Experiment 2

### Method

#### Participants

Forty undergraduates from Paris Descartes University participated in this study (19 men, mean age = 21.25 years, SD = 3.71). Each participant was randomly assigned to one of the two following experimental conditions: the “search for another path” condition (n = 20; 10 men), and the “continue in this path” condition. ANOVA and chi-squared analyses indicated that the mean ages (F(1, 38) < 1) and gender distributions (χ2 = 0.10, p = 0.75) did not differ significantly between the groups. All the participants provided written consent and were tested in accordance with national and international norms governing the use of human research participants.

#### Procedure

The procedure was similar to the one used in experiment 1 except the nature of feedback provided during the egg task. Indeed, for the participants in the “search for another path” group, the feedback provided after the generation of each idea was “search for another path” regardless of the type of idea proposed. In contrast, for the participants in the “continue in this path” group, the feedback provided was “continue in this path” regardless the idea proposed.

#### Results and discussion

To examine whether the numbers of proposed solutions (i.e., fluency) within the fixation path (fixation) and outside the fixation path (expansivity) varied according to the experimental conditions, we conducted a repeated-measures analysis of variance (ANOVA) with the experimental condition (search for another path vs. continue in this path) as a between-subjects factor and the category of solution (fixation vs. expansion) as a within-subjects factor, and we used the partial eta squared (η_p_^2^) and Cohen’s d to examine the effect size.

This analysis revealed a main effect of the solution category (F(1, 38) = 5.53, p = .02, η_*p*_^*2*^ = .13, Power = .63, see Fig 1B) that indicated that the participants provided more solutions in the fixation path (M = 5.9, SD = 3.03) than in the expansion path (M = 3.9, SD = 3.59). There was no main effect of the experimental condition (F(1, 38) < 1), nor significant experimental condition x category of solution interaction (F(1,38) < 1). These absence of effect suggested that participants do not interpret the feedback “search for another path” as meaning to be more creative and confirmed that congruent executive feedback are required to positively influence creative ideas generation.

## General discussion

The findings of the present study showing that congruent executive feedbacks increase creative ideas generation are in accordance with those of previous studies in that feedbacks in general, and more precisely executive feedbacks, can strongly influence and regulate the creative performances of individuals [24]. Moreover, these findings are consistent with those of the majority of studies that have argued that the delivery of constructive feedback can positively influence creativity [25–28] and extend previous findings by demonstrating that such constructive feedbacks can assume simpler forms, such as elementary and minimal guiding instructions (e.g., instructions such as “continue in this path” and “search for another path”). Such feedback requires minimal effort from the instructor given that he has the capacity to approximately recognize the frontier between fixation and expansion.

Our results also confirmed that fixation effects do exist in creativity and that these effects that tend to focus on usual and common ideas to solve a problem (i.e., ideas belonging to the fixation path) can be reinforced using incongruent executive feedback. This result is in accordance with those of previous studies that have demonstrated the strength of the fixation effect in creative idea generation and the difficulties of redirecting an individual toward expansive reasoning (2; 7–11).

## Conclusions

In conclusion, our results clearly demonstrate that incongruent feedback reduces individuals’ creative performances by decreasing the generation of ideas outside fixation and increasing the generation of ideas inside fixation. In contrast, congruent feedback enhances individuals’ creative performances by increasing the generation of ideas outside fixation and decreasing the generation of ideas inside fixation. Finally, the process of the generation of ideas inside fixation is much more free-flowing that the process of the generation of ideas outside fixation, which confirms that the generation of ideas inside fixation requires less effort and is more automatic and intuitive according dual-process model of creativity. As such, it is notable that these results provide new insight into research on the modeling of new forms of creative leadership from a learning perspective in which creative leaders could have an influence on their followers’ creativity level based on cognitive approaches to idea generation that involves influencing the followers’ cognitive reasoning rather than influencing other aspects related to creativity (such as intrinsic or extrinsic motivation, creativity-supportive environment, etc.) [35, 36].

## Supporting information

S1 File Supporting information files. (XLSX)
